# Prioritising time for communication in healthcare—a normative analysis

**DOI:** 10.1007/s11019-026-10364-0

**Published:** 2026-05-25

**Authors:** Joar Björk

**Affiliations:** 1 Department of Health, Medicine and Caring Sciences (HMV), Swedish National Centre for Priorities in Health, Linköping University, 581 83 Linköping, Sweden; 2 Centre for Research Ethics & Bioethics, Uppsala University, Uppsala, Sweden; 3 Department of Research and Development, Region Kronoberg, Växjö, Sweden

**Keywords:** Ethics, Priority setting, Communication in healthcare, Formal equality, Trade-offs

## Abstract

Modern healthcare faces a mismatch in terms of supply and demand which makes careful priority setting imperative. The debate on priority setting in healthcare has focussed on pharmaceuticals and other tangible medical interventions. This article instead looks at the time that healthcare professionals have at their disposal. How should healthcare professionals divide their time between tangible medical interventions and communication with patients? What communicative goals should be prioritised? This normative analysis focusses on situations which involve time consuming communication between professionals and patients, which cannot easily be carried out alongside other medical interventions or is itself the main intervention as in psychotherapy. The arguments brought forth are applicable across the healthcare panorama and apply to all healthcare professions. It is argued that there are many different communicative goals which cannot be reduced to one type. Some recommendations are given and defended regarding the priority setting of different communicative goals. For reasons of value coherence and formal equality, all patients should be given a baseline amount of time for communication and time beyond that should be prioritised based on healthcare needs. If accepted, the arguments here highlight that a substantive part of healthcare’s (time) resources should be spent on communicative tasks. Indeed, it is time that we take time for communication seriously.

## Introduction

Modern healthcare faces a mismatch in terms of supply and demand. Some of the many factors that contribute to this difficult state of affairs are increased expectations from patients and next-of-kin (Sundar 2025), increasing prevalence of chronic conditions (Polanco et al. 2025), increased numbers of expensive treatments and ambitious care regimens (Vallano and Pontes 2024), and workforce shortages (OECD/European Commission 2024). The situation makes a very strong case for careful priority setting in healthcare, so that the limited resources are spent in accordance with relevant medical and ethical ambitions in healthcare (Barasa et al. 2014; Seixas et al. 2021; Born et al. 2024).

From an ethical point of view, priority setting is about distributive justice and formal equality. Hence, the normative challenge in priority setting is to articulate which aspects should – and, crucially, which should *not* – go into deciding how to allocate healthcare resources. The practical challenge is to make sure that the relevant aspects govern resource allocation and that the irrelevant aspects are barred from influencing allocation decisions. Most ethicists agree that by virtue of formal equality the same priority setting principles should govern from top to bottom in the healthcare organisation (Hofmann 2020), as well as across different types of healthcare interventions (such as pharmaceuticals, surgery, investigations etc.) (Baltussen et al. 2016). This way, like will be treated alike regardless of at what level of organisation the decision is made and which kind of intervention is suitable.

While priority setting is relevant in all types of healthcare organisations, some publicly financed healthcare systems have taken a leading role in formulating priority setting frameworks (Sabik and Lie 2008; Glassman and Chalkidou 2012; Seixas et al. 2021). Some countries have developed guidelines for healthcare priority setting. Although there are differences between such guidelines, due not only to normative differences but also to nationally contextual features, a recurring theme is that the patient’s *healthcare need* is one of the most important aspects to govern priority setting (Baltussen et al. 2016; Norheim 2016; Seixas et al. 2021).

Much normative and descriptive work in priority setting has focussed on the priority setting of, for instance, pharmaceuticals and ICU beds (Seixas et al. 2021). However, one area which has been less discussed is priority setting challenges regarding *provider-patient communication*^1^. The aim of this article is to provide a background to communication-related priority setting challenges, as well as provide some normative proposals.

Under the umbrella of communication-related priority setting challenges are (at least) two different challenges. First there is the trade-off between different kinds of communication, i.e. what communicative *content* should be prioritised (and why). Second, there is the trade-off between communication and all other tasks to which healthcare personnel might devote their time (for instance: ordering CT scans, performing surgery, or administering intravenous antibiotics)^2^. Both of these challenges will be given due attention in this article.

To ensure that all readers are aboard with the kind of priority setting challenges that will be analysed here, the following brief vignettes are provided as examples:

VIGNETTE 1: *Side effects or patient’s own questions?*

After having performed a thorough examination of a worried patient, the GP realises it will be impossible, within the time remaining, to notify the patient that there were no signs of a tumour but the blood pressure is high, suggest anti-hypertensives, inform about the potential medical benefits and side effects, and answer at least a few of the many questions the lady is sure to have.

VIGNETTE 2: *Replacing the needle or hearing more about the patient’s choice?*

Nearing the end of a long day, the ward nurse realises she must choose between replacing the needle in one patient’s implanted venous access device, and going back to chat with another patient, who previously expressed a desire to explain in further detail why he had opted out of the recommended chemo-therapy treatment.

## Methodology

In terms of priority setting and fair distribution of resources, the most relevant aspect of communication is the *time* that healthcare professionals (abbreviated: HCP) use to communicate. Other aspects, such as costs for communication interfaces like eye movement computers or other speech aids, will not be considered here. Henceforth, Time for Communication will be abbreviated as TfC. By communication is intended, here, only the forms of communication between HCP and patients which can present priority setting dilemmas of the relevant kind. Hence, communication which is very little time consuming, can easily be carried out alongside other medical interventions or which is itself the main intervention (as in psychotherapy) will be ignored here. On the other hand, it must be noted that communication is seen, here, as much more than merely “providing information”, and includes the whole range of communicative interchanges that may take place between HCP and patients (Coyne and Dieperink 2025).

As for clinical context, priority setting challenges involving communication can be seen at all levels of care in a publicly financed healthcare system, and is thus not confined to, for instance, hospital care. Neither is it confined to any one professional category, and hence talk will be of HCP rather than e.g. physicians. There is a further priority challenge which concerns time spent on talking *to other HCPs* (in rounds, consultations, multi-disciplinary team meetings etc.) but as these have partly different goals (education etc.) they will be left out of the picture here.

The article will proceed as follows. First, I will propose that the potential goals related to providing TfC are multidimensional, and that an adequate normative analysis of prioritising communication in healthcare requires a rich understanding of these values. Second, I will attempt to provide recommendations for the priority-setting of different communication-related goals in situations where there is enough TfC to strive towards some, but not all, communication-related goals. Third, I will propose that the values related to TfC are non-inferior to the values related to other forms of medical tasks, and hence it should be accepted that TfC sometimes crowds out time for other tasks. Last comes the proposal that some TfC should be distributed according to patients’ healthcare needs. A conclusion wraps up.

### Multidimensionality of communication values

Thinking about priority setting means thinking about the value gains which may be had, by patients, from different possible ways to (re)allocate resources in healthcare. In the case of TfC, the potential gains of allocating much time to communication in healthcare are heterogenous (De Haes and Bensing 2009; Street Jr et al. 2009). With no intention of being comprehensive, the first column of Table 1 presents some potential values which may be gained by allocating ample time to TfC (values in no particular order).

**Table 1:** Potential values of TfC, their potential beneficiaries and the relation between these values and ethical principles according to Beauchamp and Childress

Potential value	Value is potentially relevant in the Vignettes?	Who is the potential beneficiary of the value?	Which Beauchamp & Childress’ principle is the value most related to^1^?
Securing informed consent to treatment (Beauchamp 2011)	Vignette 1	Individual patient	Autonomy
Helping the patient understand his/her medical situation in a more general sense than that required for informed consent^2^ (Grover et al. 2022)	Vignette 2 and Vignette 3 (see pg 10)	Individual patient	Autonomy
Involving the patient in his/her care in a more general sense than that implied by, for instance, informed consent (Sharp et al. 2016)	Vignette 2 and Vignette 3 (see pg 10)	Individual patient	Autonomy^3^
Improving the patient’s adherence to suggested medications (Aremu et al. 2022)	Vignette 1	Individual patient	Beneficence
Allowing patients space to ask the questions that they find pressing (General Medical Council 2020)	Vignette 1 and Vignette 3 (see pg 10)	Individual patient	Autonomy
Lowering the risk of misuse and side effects of treatments by making the patient aware of risks and signs of side effects (Berkman et al. 2011) in a more specific way than that implied by informed consent	Vignette 1	Individual patient	Non-maleficence
Gathering information about patient’s preferences to be able to tailor care according to these (Beauchamp and Childress 2019)	Vignette 2	Individual patient	Autonomy
Creating trust (Hall et al. 2001)	Vignette 1, Vignette 2 and Vignette 3 (see pg 10)	Individual patient	Beneficence
Connecting as humans (Makoul et al. 2024)	Vignette 2	Individual patient	Beneficence
Providing emotional support^4^ (Larson and Yao 2005; Pincus et al. 2013)	Vignette 2	Individual patient	Beneficence
Saving TfC later by spending TfC now (Cappella and Street 2024)	Vignette 1	Individual patient and/or other patients	Justice^5^
Levelling power imbalances (Richards et al. 2013; Gulbrandsen et al. 2016; Sundler et al. 2020)	Vignette 2	Individual patient and/or population	Justice^6^
Increasing the general level of health literacy in the population^7^ (Grover et al. 2022)	–	Population	Justice^6^
Improving the publics’ understanding of the healthcare system and correcting misunderstandings about health care^8^	–	Population	Beneficence
Gathering information as part of an adverse event review (McQueen et al. 2022)	–	Population	Beneficence/Non-maleficence

^1^I am aware that some values could be considered “most related” to different, or several, principles depending on your interpretation of Beauchamp & Childress’ framework (Beauchamp and Childress 2019). However, the precise exegesis of PBE is of less importance here and the reader can simply insert his/her favourite interpretation

^2^Readers may object that several items on this list could be seen as aspects of the communication leading up to (potentially) informed consent. It makes no difference to the thrust of my argumentation whether some aspects are subsumed under the rubric “informed consent” or not. The argument is that there are several, non-reducible goals in provider-patient communication regardless of how one chooses to group or label them

^3^At least with a ”broad” understanding of autonomy (Entwistle et al. 2012)

^4^As purely therapeutic conversations are not in focus here the ”emotional support” intended is of the everyday kind that can be provided by HCP without any specific psycho-therapeutic education

^5^At least if the TfC saved could benefit other patients

^6^If those most likely to benefit belong to the least advantaged groups in society

^7^This does not include situations where health education is the main intervention

^8^Potential misunderstandings about health *care* (as opposed to health *simpliciter*) may involve, for instance, wide spread rumours about vaccinations (Lee et al. 2022)

Any potential value associated with (good) communication, obviously, provide potential arguments in favour of providing ample TfC. Interestingly, however, the heterogeneity of these values makes it hard to subsume them all into *one* principle such as “the value of communication”. In the right-most two columns of Table 1 is an attempt to spell out who may be the beneficiaries of different values, and which of Beauchamp & Childress’ influential four principles most relate to the individual values (when applicable). As can be seen from the table, the values differ in many aspects. Yet other aspects of difference include the fact that some values are largely or entirely intrinsic (notably those related to autonomy) whereas others are instrumental; and the fact that some communicative tasks can be scripted *ex ante* (consider for instance the use of different forms of standardised script in securing informed consent (Glaser et al. 2020; Chimonas et al. 2023) whereas others must be invented at the spot (such as “Connecting as humans”). In light of this, the first proposal of this article is that *the potential values are so different that they cannot meaningfully be reduced to one single value*. There are two independent arguments to buttress this claim. First, the communicative *strategies* used may differ depending on the goal (Denniston et al. 2017): informing about aims and side effects of treatment can take the teacher-pupil format, whereas aspects having to do with renegotiating roles demand an anti-authoritarian attitude from HCP, and values such as “Allowing patients space to ask the questions that they find pressing” require most of all the competency to ask short, focused questions which open up the space for patients to talk, coupled to strong listening skills. Second, there may be a risk of value conflicts between these values^3^. For instance, a strong focus on potential side effects may compromise understanding, adherence and/or trust (Barker and Faasse 2023). Hence, to have a rich understanding of the ethical context of priority setting conflicts involving communication, a non-reductionist view of the potential values of communication is necessary.

### Prioritising among communication-related values

There may be situations where HCP have sufficient TfC in a particular situation to strive for *some*,* but not all* of the potential goals listed above. This raises the priority setting question: which goals should be prioritised? As a first disclaimer, it must be stressed that healthcare is so heterogeneous that it is likely impossible to develop clear guidelines which apply equally to all parts of healthcare. This being said, some general normative advice may nonetheless be provided.

In situations which demand informed consent, *spending the TfC necessary for securing informed consent should be the first priority*^4^,^5^. However, it must be noted that “first priority” does not imply “most of the TfC” but rather “sufficient time to secure informed consent”. Hence, the proposal is that in most clinical situations, it is necessary to provide a baseline level of TfC to secure informed consent. This follows from the strong normative weight placed on informed consent in modern healthcare (Beauchamp 2011), and it may further be argued that most other aspects of provider-patient communication hinge on whether the patient has consented and thereby accepted to partake in the process which is to follow (Spriggs 2023).

There is a further complexity, of relevance for priority setting and formal equality, that must be noted here: the TfC necessary to secure informed consent is likely to differ between patients. There are several reasons for this, including the fact that some patients may have borderline decision making capacity in the sense that provided ample TfC they will be able to partake in medical decision making (General Medical Council 2020). That healthcare should be ready to expend more resources on weak groups (such as those that need much support to be able to partake in medical decision making) is commonly accepted as a consequence of caring about equity (Daniels and Sabin 2008; Norheim et al. 2014; Yamin and Norheim 2014).

Turning now to the other autonomy-related values (see Table 1 above) it seems that ethical coherence demands that, if the priority for TfC for securing informed consent is accepted, then the other autonomy-related goals must also have high priority. Indeed, the reason why we care about informed consent is (or better: *should* be (O’Neill 2003) because we care about patient autonomy. To insist that we should care about patient autonomy but *only* in the sense that patients should provide informed consent and not, for instance, get a chance to ask at least some of the questions they find pressing is an odd stance which lacks theoretical support from the literature on autonomy. Hence, *for the same reason that we prioritise spending TfC on securing informed consent we should devote some more TfC to the other autonomy-related values also.* Unlike TfC for informed consent, which is easy to delimit, the TfC for other autonomy-related values is less easy to specify. Indeed, it is often easier to know when somebody has been provided enough time to give their informed consent than to ask their most pressing question(s). To the best of the author’s knowledge there is no accepted value hierarchy for the different communication goals related to autonomy. Hence, a preliminary rule of thumb may be that the baseline TfC for autonomy-related values should be equal to the time necessary to secure informed consent + equal TfC for the other autonomy-related values. The suggestion to spend equal time on other autonomy-related goals as on time to secure informed consent is of course arbitrary – some other ratio could have been possible – but reflects a lamentable omission in the scholarly literature on autonomy.

It is time (!) now to turn to the other values. There is a group of values where there the priority-setting of TfC is highly dependent on contextual features. This is true of TfC related to beneficence and non-maleficence (see Table 1 above). In some situations, spending TfC to discuss how to lower the risk of side effects of a treatment may make the difference between a safe treatment and a very risky treatment. If the potential effects of a treatment are very beneficial, then spending TfC to improve adherence should be highly prioritised. Similarly, the values of “Providing emotional support” and “Saving TfC later by spending TfC now” depend on whether the patient is in emotional distress and on whether spending TfC now can be expected to prevent a bigger need for TfC in the future. As for the value of “Creating trust”, contextual features again play a large role in determining how TfC should be prioritised. Evidence suggests that as long as the HCP-patient interaction goes smoothly, trust does not need to be consciously “created” but rather follows as a side effect of all other signs of appropriate professional conduct (Lerch et al. 2024). In these situations, then, it seems that no or very little TfC must be spent with the specific purpose of creating trust. If, however, there are signs that there is manifest dis-trust or high risks of dis-trust, then “Creating trust” would seem to be a necessary prerequisite for most or all other healthcare work. Hence, *the priority setting of TfC related to Improving adherence*,* Lowering the risk of side effects*,* Providing emotional support and Creating trust depends on contextual features*.

I propose that the remaining potential values of TfC should *not* be given high priority in the allocation of time in individual provider-patient interactions – for different reasons. When it comes to “Connecting as humans” and “Levelling power imbalances” these are (largely) a matter of showing certain attitudes (humility, interest, empathy etc.). In other words, they are not a stand-alone communication task and so are not expected to (generally) compete for TfC^6^. As for the values of increasing health literacy and understanding of the healthcare system in the population, these should likewise have low priority *in the individual provider-patient encounter*, as they can easily be delegated to other actors and/or other forums^7^. Indeed, if HCP had all the time in the world there would be nothing to stop them from providing health education and helping patients understand the healthcare system beside all their other tasks. Again, however, the premise of this article is that HCP do not have all that time and hence priority decisions must be made. Finally, conducting thorough adverse event reviews is very important task but one which is generally carried out by others than those who are directly responsible for patient’s care, and hence the TfC for adverse event reviews does not compete with care-related TfC. This is not to say, however, that there is no priority setting dilemma between care-related TfC and TfC related to the latter values. Instead, this is a priority setting dilemma at a higher level of healthcare provision, or even between healthcare and, e.g., the education system (when it comes to increasing health literacy).

The suggestions made in this section are summarized in Table 2.

**Table 2:** Some normative recommendations regarding intra-TfC trade-offs

Highly prioritised	Contextual features determine priority	Not prioritised as direct goals or as goals for the individual provider-patient interaction
Involving patient in care	Improving adherence	Levelling power imbalances
Helping the patient understand the medical situation	Lowering risks of misuse and side effects	Connecting as humans
Gathering information about preferences	Providing emotional support	Increasing general health literacy
Securing informed consent	Saving TfC later by spending TfC now	Improving understanding of the healthcare system
Allowing free space for patient to ask	Creating trust	Adverse event reviews

### Prioritising between TfC and other forms of medical tasks

The discussion has so far focussed on *intra*-TfC trade-offs, i.e. trade-offs between different forms of communicative content (see also Vignette 1). However, the more challenging trade-offs of the kind exemplified in Vignette 2, i.e. trade-offs between TfC and spending time on *other forms of medical tasks* such as ordering CT scans, performing surgery, or administering intravenous antibiotics. In regard to this kind of trade-off, I propose that *the baseline level of the highly prioritised TfC content (mentioned in the previous section)* ***must*** *be provided*,* even though this may crowd out some time for other forms of medical tasks*^8^.

There are several ways to defend this proposal. The first is to insist that as a matter of coherence, we must substantiate our claims to care about autonomy in general and informed consent in particular by giving sufficient TfC for actions related to these goals *even in comparison with other but non communication based medical tasks*^9^. The other is to hark back to the “principlistic equality” in Beauchamp and Childress’ work, whereby it is clearly stated that the four principles have prima facie status and have, in the abstract, equal weight (Beauchamp and Childress 2019; Scotch et al. 2025). This implies that autonomy-related tasks should not *as a rule* stand back in the challenge for time compared to, for instance, beneficence-related non-communicative tasks such as performing surgery. The third is to return to the discussion about trust, above, where it was argued that under normal circumstances trust follows as a side effect of appropriate professional conduct. It seems reasonable to assume that one aspect of appropriate professional conduct is that some TfC is always provided and, conversely, that purely medical tasks are not allowed to crowd out all TfC^10^. The implication, then, is that we must continue to provide *sufficient* TfC because otherwise, we would need to provide *plenty* of TfC to create trust.

Importantly, I propose that the highly prioritised TfC content must be provided independent of whatever other priority the patient’s medical situation has. Turning back to the argument in the previous section, this does not imply that it is highly prioritised – in all cases – to provide *ample* TfC, but rather that a baseline level of TfC should be provided to all patients to live up to the most fundamental autonomy obligations like involving the patient in her care, securing informed consent, and allowing her at least some space to ask some pressing questions of her own choosing. In addition, contextual features may sometimes make some of the other values highly prioritised, as explained in the previous section.

### What about patients’ (healthcare) needs?

The principle of formal equality, hailing back to Aristotle, states that like should be treated alike, and unlike should be treated unlike (Sandman 2023). To set priorities in a fair way thus involves identifying whether patients (or patient groups) are alike or unlike in the morally relevant sense, as only unlike patients (or groups) should be prioritised differently. As previously mentioned, there is near consensus that *need* (in some sense) is a morally relevant aspect for priority setting, such that patients with a higher degree of need should have higher priority. The author sees no compelling reason to question the relevance of need as a morally salient aspect in priority setting. But as the careful reader has noticed, the proposal so far contains no needs-sensitive aspect. Indeed, the gist of the argument so far has been to claim that *all* patients should be awarded a baseline of TfC *independent* of need. Hence, there is a need to question whether there should also be a needs-sensitive aspect to the provision of TfC. As the author can see no compelling reason why TfC should be exempted from the general rule that healthcare resources should be prioritised at least in part according to need, the proposal here is that *some TfC should be prioritised according to need.* As all patients must be offered an equal baseline of TfC, the needs-based TfC will determine the level of attempted goal fulfilment, which should be higher in patients with greater needs so as to satisfy the desiderata that those with greater needs should get a greater share of the resources. In the clinical setting, this should mean that *ample* TfC should be reserved for those patients whose needs are great^11^.

There is a long discussion in healthcare priority setting on the topic of what, exactly, is intended by “needs” in the sense relevant for priority setting (Gustavsson 2017). The question is pressing here as it seems possible that two patients with similar *healthcare* needs may have different TfC needs^12^. Consider the following vignette:

VIGNETTE 3: *Explaining in detail?*

The physician has recommended the surgical excision of a benign tumour and the 24-year-old patient has agreed. However, the patient wants to know “all the reasons” for the physician’s recommendation including why other forms of treatment were not recommended. The appointed time runs out and the physician offers an additional 20-minute long appointment before surgery. The patient brings three sheets of closely printed script with his questions, and the physician realizes that answering all would take at least three times as long as she has scheduled.

The patient in vignette 3 may be said to have a great communicative need – he clearly desires a lot of information from his physician. Harking back to Table 1, there is a chance for the physician to help this patient understand his medical situation in a more general sense than that required for informed consent (as he has already consented). However, his desire for communication is out of proportion to his healthcare need, as the benign tumour is a harmless condition. How should TfC be prioritised for this patient?

First, it should be noted that cases like Vignette 3 are likely unusual. In most medical situations we may expect that healthcare needs and communicative needs follow each other. This is reflected in the routine practice to offer counselling and chaplaincy services to palliative care patients, as these have great healthcare needs and (often) great communicative needs. But when, as in Vignette 3, the healthcare need and the need/desire for communication come apart it seems reasonable to go with the healthcare need. Here I follow Lars Sandman and Erik Gustavsson who note that people may have all sorts of needs but that their *healthcare needs*, more specifically, are the ones relevant for priority setting purposes (Gustavsson and Sandman 2015). This does not negate the fact that some patients may need more TfC to realise the same value(s). In that sense “TfC need” may indeed be relevant. In order to honour formal equality, we should be prepared to devote more TfC to, for instance, patients with another language or patients with cognitive disorders, who may need more time and more support to process information (Sandman and Gustavsson 2017; Wieringa et al. 2024). This is also the normative stance taken in many countries’ policy guidelines. For instance, UK’s General Medical Council write: “All patients have the right to […] the time and support they need” (General Medical Council 2020)^13^. Differently put, patients may be given more TfC to reach ordinary TfC goals, and patients with bigger healthcare needs may be given more TfC to track their healthcare needs, but patients may with *higher set TfC goals* should not routinely be awarded more TfC.

## Conclusion

In this article, two forms of trade-offs regarding how HCP spend their time have been investigated. The first regards which forms of communication HCP should prioritise, and the other regards how TfC should be prioritised in comparison to other, non-communicative tasks in healthcare (such as ordering CT tests etc.).

In response to these challenges, I have proposed that there are multiple, non-reducible values at stake by providing TfC, that some of the values are more important than others, that TfC must be allowed to crowd out some non-communicative healthcare tasks and that some TfC must be given equally to all patients and that some TfC should be distributed according to need (more specifically: *healthcare* need). With all of these puzzle pieces in place, it becomes obvious that one reason for the complexity here is that healthcare strives for (at least) two goals at the same time: improved health *and* patient autonomy. My proposals try to make good of this complexity.

If accepted, the joint impact of all my proposals is that healthcare must allow a substantive chunk of HCPs’ available time to be consumed by communicative tasks. Indeed, the time that should be prioritised for communication is likely much greater than what is current practice in most medical field.

Some questions remain. First, this article presents the general normative framework of the argument, but more could be said about the details. Second, an idea which has merely been hinted at in the present work is that some communication tasks could perhaps be externalised to new actors. Another article delving into this issue is forthcoming^14^. Fourth, very little is known about how HCP understand and deal with real-world TfC priority setting challenges in the clinic, or how patients respond to this. Although empirical studies cannot solve the normative challenges, more knowledge regarding for instance HCPs current trade-off strategies could help present normative recommendations in a context-sensitive fashion. In a nutshell: it is time that we take time for communication seriously.

## Footnotes

1 Obviously, there are also challenges involving communication with next-of-kin. The general line of reasoning in this article will apply to communication with next-of-kin also, and for this reason “patients” is used as a shorthand for “patients and/or next of kin”. There is a *specific* trade-off challenge concerning whether time for communication should be spent with the patient or with the patient’s next-of-kin, which will not be explored here.

2 As pointed out by an insightful reviewer, there may be further ethically relevant trade-offs involving time for communication, such as the trade-off between time spent in actual communication and time spent *learning communication skills.*

3 As is the case in any situation with plural and heterogenous values.

4 There is a lively debate as to which situations actually demand informed consent (Agich 2003; Manthous et al. 2003; Dunn et al. 2019). This is not the place to take a stand in that debate. It is enough to state that the discussion here concerns such situations where informed consent is demanded.

5 Or, for that matter, informed refusal (Bernat and Peterson 2006).

6 This may be a simplification. It could be that communication which expresses the desired attitudes takes, on average, longer time than communication which lacks these attitudes. One standout situation is when the patient lacks decision making capacity, and medical treatment is carried out without the patient’s consent (as in legally mandated coercive care, or per instructions in an advance directive). In such a situation it is often necessary to spend much TfC on “connecting as humans” and “creating trust”. Hence, there could be a priority challenge against other communication goals and/or other general medical goals. At the same time, in the interaction with a patient who lacks decision making capacity (and the capacity to consent to treatment) many of the other communication goals become less relevant.

7 As pointed out by one reviewer there may be situations where delegating certain health literacy related tasks – for instance, discussing vaccine hesitancy – may result in lower trust and lower chances of communicative success. Hence, as with other normative suggestions, a keen eye to the specifics of the context, is mandated.

8 The highly prioritised TfC content is given in the left column of Box 2.

9 Reis-Dennis seems to share this view when he claims: ”The right of informed consent is treated as absolute in Western science and medicine. Even the suggestion that clinicians and scientists should weigh and balance other moral principles against this basic entitlement would shock many practicing physicians, nurses, and researchers” (Reis‐Dennis 2023).

10 Recall that we are discussing, here, communication which goes beyond that which can easily be carried out alongside other medical interventions or which is itself the main intervention.

11 Obviously, TfC is not simply doled out to patients but offered to them. Patients regain their autonomy-based right to refuse TfC. Hence some patients who have a great need will not be given great amounts of TfC for the simple reason that they do not wish it.

12 The discussion here is different from the one in footnote 15 as we are now considering patients who all have full decision-making capacity.

13 Turning the question on its head yields a trickier challenge. Imagine we have two patients with the same healthcare need. However, providing X minutes of TfC will satisfy all normal communicative obligations for the first patient, but provide the second patient with supernormal levels of all the values indicated in Table 1 so that the outcome of TfC is much greater in the second patient. This is analogous to Robert Nozick’s utility monster (Nozick 1974).

14 Working title: The autonomy ought-is gap and its potential solution – a call for “autonomy consultants” in modern healthcare (co-author: Anna Hirsch).
